# Ventricular tachycardia ablation in a patient with Ehlers-Danlos syndrome

**DOI:** 10.1016/j.hrcr.2021.12.007

**Published:** 2021-12-13

**Authors:** Peter Calvert, Gavin Chu, Archana Rao, Dhiraj Gupta, Vishal Luther

**Affiliations:** ∗ Liverpool Heart & Chest Hospital, Liverpool, United Kingdom; † Liverpool Centre for Cardiovascular Science, University of Liverpool, Liverpool, United Kingdom; ‡ Department of Cardiovascular Sciences, University of Leicester, Leicester, United Kingdom

**Keywords:** Ventricular tachycardia, Ablation, Ehlers-Danlos syndrome, Ripple mapping, Connective tissue disorder

**Key Teaching Points**

• When performing endovascular procedures, all comorbidities should be considered in terms of risk vs benefit to the patient. This may include rare conditions such as Ehlers-Danlos syndrome or other connective tissue disorders, which may affect vascular stability, risk of bleeding, and wound healing.
• A stepwise approach to intervention in such patients is advised, as detailed in our manuscript. This should include thorough imaging, multidisciplinary team discussion, and a careful, individualized consent process.
• Ventricular tachycardia ablation can be highly effective at suppressing scar-related life-threatening arrhythmia and can be safely undertaken in those with mild connective tissue disease, such as our patient. More severe disease may require more a more conservative approach.

## Introduction

Ventricular tachycardia (VT) is a well-recognized complication of postinfarct-related scar. Catheter ablation is a guideline-recommended therapy in patients with frequent VT episodes. Ablation aims to target regions of slow conduction within ventricular scar that support reentry.^1^

Patients with prior myocardial infarction often have multiple comorbidities. While most will not interfere with a planned ablation procedure, occasionally one may encounter a condition that requires some forethought preoperatively. Ehlers-Danlos syndrome (EDS) is an inherited connective tissue disorder that can affect multiple organs, including the heart and vasculature.^2^ Little is known about the safety of undertaking cardiac procedures in patients with EDS. We describe a case of a patient with EDS who required ablation for postinfarct VT and present an algorithm of best practice when planning cardiac procedures in such patients.

## Case report

A 63-year-old man presented to hospital with breathlessness. His history included inferior myocardial infarction and impaired left ventricular (LV) function (ejection fraction 33%), with transmural scarring of the basal-to-mid inferior and inferolateral walls. He had a primary prevention implantable cardiac defibrillator (ICD). He was NYHA class II on reasonable heart failure therapy (bisoprolol 10 mg, perindopril 8 mg, and eplerenone 25 mg, all once daily). He had EDS, though he was not under specialist review.

**Figure 1:**
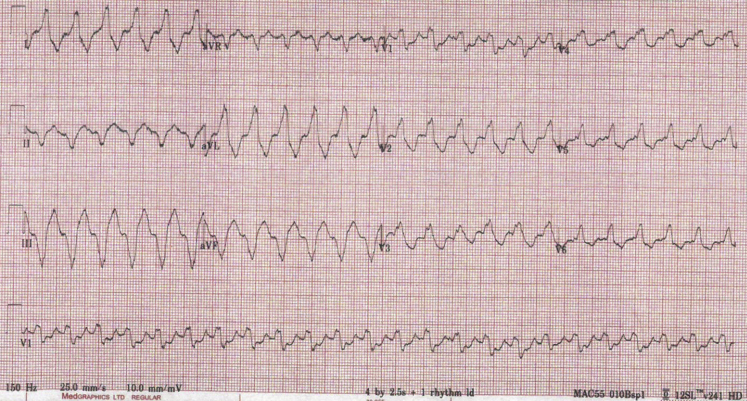
Twelve-lead electrocardiogram demonstrating monomorphic ventricular tachycardia: right bundle branch block morphology, superior axis, positive concordance in the precordial leads, and positive QRS in lead I, suggestive of a basal left ventricular inferoseptal exit from the known scar.

An electrocardiogram revealed he was in a tolerated monomorphic VT (Figure 1). The morphology was suggestive of a basal LV inferoseptal exit from the known scar. The rate (143 beats/min) was below the programmed monitor and therapy zones of his device. A lower therapy zone was programmed that offered antitachycardia pacing (ATP), and this promptly restored sinus rhythm.

This was the patient’s first documented episode of VT. He reported several recent similar symptom episodes for which he did not seek medical attention. Following device reprogramming, a remote interrogation 1 week later revealed multiple asymptomatic episodes of VT, all of the same cycle length and with identical electrogram morphology. These episodes all terminated with the first burst of ATP. The patient was referred for an electrophysiology opinion to discuss VT ablation.

Options discussed included conservative management (ATP adjustments alone), antiarrhythmic medication (amiodarone), and catheter ablation, which was felt likely to be successful given the presence of a well-defined endocardial scar and single well-tolerated VT morphology.

The history of EDS was explored in more detail. Both the patient and his daughter were known to have EDS, consisting of a loss of dentition, mild skin elasticity, and slow wound healing. No genotyping had been undertaken. His exercise tolerance was limited by sciatica, for which he used a walking stick. He had no known history of cardiovascular involvement, having been previously investigated with cardiac computed tomography (CT) and magnetic resonance imaging (MRI). He had structurally normal valves. There was no personal or family history of vascular aneurysm or dissection and he had undergone previous operations without complication. Importantly, a previous CT coronary angiogram had revealed a normal-caliber ascending aorta. There was diffuse, significant disease in the right coronary artery and mid circumflex; the likely culprit of his infarct. Repeat invasive coronary angiography was avoided, given the absence of anginal symptoms and the perceived additional risk that arterial access posed in EDS.

An electrophysiology multidisciplinary team (MDT) discussion took place to explore the potential procedural risks that could be encountered. The consensus was that ablation would be feasible, but that it would be preferable to avoid arterial access and the retrograde aortic approach given the potential for arterial fragility and risk of aortic dissection. The use of transesophageal echocardiography (TEE) to guide transseptal puncture was advised, given that the septum might be more elastic than usual.

After discussing with his family, the patient opted for catheter ablation on the grounds of avoiding potentially toxic long-term antiarrhythmic medication. Furthermore, driving was essential in the patient’s personal life and he wanted the most effective therapy possible to prevent future VT episodes.

The patient was scheduled for VT ablation within 3 weeks. Antiarrhythmic agents were avoided preablation to facilitate intraoperative VT induction. On the day of the procedure, device interrogation revealed 32 episodes of VT since his first assessment, all well tolerated and terminated with ATP. The procedure was performed under general anesthesia, both for comfort (as was the patient’s preference) and to facilitate TEE.

Under ultrasound guidance, 3 7F sheaths were placed in the femoral vein. One of these was up-sized to an 8F Agilis sheath (Abbott Cardiovascular, Plymouth, MN). Transseptal puncture proved challenging owing to lipomatous hypertrophy of the interatrial septum seen on TEE. The fossa was not excessively elastic and was eventually crossed without complication. The LV was mapped using CARTO 3D navigation (Biosense Webster Inc., Irvine, CA) and a multipolar PentaRay catheter (Biosense Webster Inc.). A dense bipolar voltage map confirmed homogenous inferior/posterior “aneurysmal” scar (<0.5 mV).

**Figure 2:**
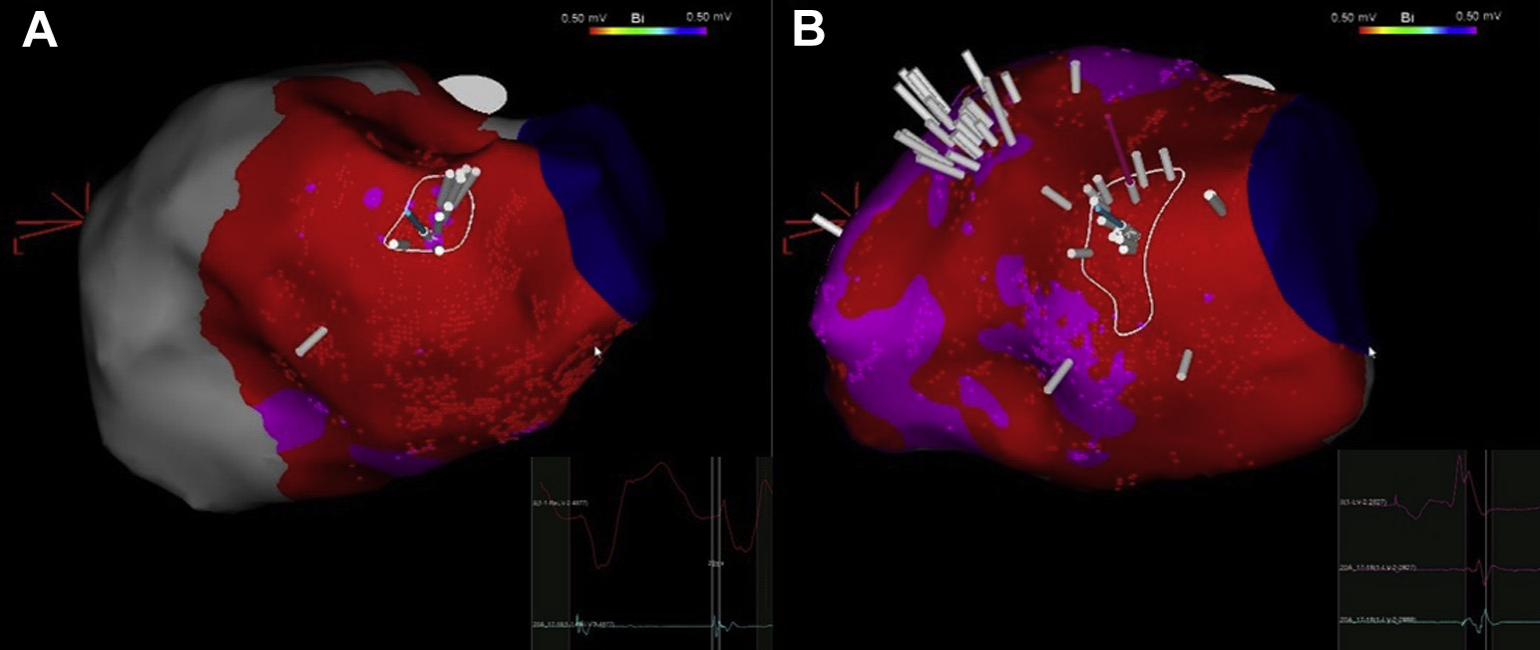
Modified posteroanterior view of the left ventricular inferior wall scar. **A:** Ripple map in ventricular tachycardia—the white ripple bars are seen to emanate from a focal point in the basal inferior wall; the local electrogram here revealed presystolic potentials emanating from scar, suggesting the rest of the circuit takes an epicardial course. **B:** Ripple mapping in sinus rhythm shows late systolic potentials co-locating within the same region. (Also see Supplemental Video.)

Programmed VT stimulation induced 2 VTs: the clinical VT seen previously and a second, more rapid and poorly tolerated rhythm exiting from the inferolateral aspect of the scar, requiring external DC cardioversion. A ripple map of the clinical VT revealed presystolic activity underneath the inferior mitral annulus (Figure 2A) that co-located with the latest potentials as seen on the ripple map in intrinsic rhythm (Figure 2B and Supplemental Video). Given the presence of VT morphologies exiting both sides of the scar, extensive substrate ablation was undertaken using a contact force–sensing irrigated ablation catheter (SmartTouch^TM^; Biosense Webster Inc.). Ablation was undertaken at 40 W for approximately 45 seconds per lesion to homogenize the entire channel of late ripple activation, targeting noncapture at 10 mA – 2 ms along the ablated sites (ablation tags available in the Supplemental Video). This resulted in complete VT noninducibility at both 600 ms and 400 ms drives with 3 extrastimuli to refractoriness.

The patient made an uneventful recovery. No access site complications occurred. He was commenced on a 2-month course of apixaban 5 mg twice daily; this is our standard practice, although evidence has been anecdotal until the recently published STROKE-VT trial, which demonstrated superiority of direct oral anticoagulants over aspirin for 30 days of follow-up.^3^ One month postablation, no further VT episodes were recorded and following discussion with the UK driving license authorities, the patient was allowed to drive. Six months later, ICD interrogation confirmed no further episodes of VT.

## Discussion

Catheter ablation is of proven efficacy in reducing VT episodes in patients with postinfarct scar. To our knowledge, this is the first report to describe VT ablation in a patient with EDS.

EDS is an inherited connective tissue disorder that can affect multiple organs. There are at least 13 subtypes.^4^ Common manifestations include easy (or spontaneous) bruising, skin hyperextensibility, joint hypermobility, and internal organ / vascular fragility. These features may be overlooked by both patients and clinicians leading to delays in diagnosis. From a cardiovascular perspective, mitral valve prolapse and aortic root dilatation are associated with EDS. This is particularly relevant in the classic (type 1) and vascular (type 4) forms of EDS, and recognition of these associations is of utmost importance if invasive procedures are planned. Type 4 (vascular) EDS is a rare form (1 in 90,000) that carries a poor prognosis (50% mortality by age 48) and is associated with vascular dissection and hollow organ rupture.^5^ Clearly, in this situation it is desirable to avoid vascular instrumentation if possible.

Other than case reports, there is minimal literature available on the safety and feasibility of cardiac (or indeed any) interventional procedures in EDS. The most relevant cardiac case report was of a 59-year-old EDS patient (of unknown subtype) who presented with syncope and recurrent VT.^6^ A coronary angiogram was complicated by a femoral hematoma. An ICD was planned—the team avoided a subcutaneous device on the grounds of skin laxity and likelihood of device migration; however, the implanted transvenous ICD was complicated by delayed right ventricular apical lead perforation and pericardial effusion that required sternotomy (itself complicated by pneumothorax and subcutaneous emphysema) to explant the system. Concerns about myocardial fragility led to a decision not to replace the device. This patient also had atrial arrhythmia and TEE-guided cardioversion proved problematic owing to difficulties advancing the TEE probe along the tortuous and flexible esophagus. This patient likely had a more malignant form of EDS.

There is a single case report of successful and uncomplicated slow pathway ablation in a patient with type 4 (vascular) EDS.^7^ In this case, to minimize venous access and intracardiac instrumentation, a single-catheter approach with a magnetic guidance system was utilized (stereotaxis), which employs a softer catheter tip.

An expert review article by Ghali and colleagues^4^ advised against surgical and endovascular procedures in the more malignant phenotypes of EDS (ie, vascular EDS) where at all possible, as the risk of complication is high. We suspected that our patient had a mild phenotype of EDS. We carefully reviewed the prior cardiac CT and MRI of our patient, which revealed trivial mitral regurgitation and normal caliber of the aorta. He had no history of vascular dissection or organ rupture. He had previously tolerated general anesthesia, had an uncomplicated cholecystectomy in the past, and had no issues with wound healing or ICD lead implantation when deployed in the right ventricular apex. Our patient tolerated TEE and an aggressive ablation protocol targeting noncapture without complication. Thus, anecdotal case reports such as ours suggest that those with a milder EDS phenotype may be at lower risk of complications. Despite this, we remained concerned about the risk of arterial access and avoided invasive angiography or ablation via the retrograde aortic approach.

We had anticipated encountering an elastic interatrial septum, potentially complicating transseptal access; however, this was not observed. In retrospect, other than case reports describing atrial septal defects in EDS^8^ there is no literature around hyperextensible septa. Interestingly, a case-control comparison of 33 EDS patients found no significant echocardiographic difference between EDS and control groups.^9^ We used TEE to guide the transseptal puncture without complication. In hindsight, esophageal tissues may also be fragile and there is an increased risk of iatrogenic esophageal perforation. An alternative would be intracardiac echocardiography; however, this would require a further instrument passed into the venous system, increasing the risk of vascular complications.

We used ripple mapping to guide our ablation set. CARTO ripple mapping displays every electrogram deflection as a perpendicular moving white bar on the map, the height of which corresponds to the electrogram bipolar voltage through time. The map can be played over a bipolar voltage map, allowing for simultaneous activation and scar displays. In our experience, late ripple bar activation (post-QRS) corresponds with late potential activation through scar and co-locates with the VT isthmus.^10^ A demonstration of this is shown in the Supplemental Video.

### Stepwise approach to electrophysiological intervention in EDS

Firstly, consider how much is known about the patient’s EDS. Is this a confirmed diagnosis? Do they know their subtype? Where a subtype is not known, the benefit of investigating this should be weighed against the urgency of their interventional procedure and the likelihood for harm, which may arise owing to delays in intervening in certain cases, but may equally arise from vascular damage where the subtype is not established.

Specific consideration should be given to the following (list adapted from reference^11^):

• Wound healing, which may be impaired in EDS and result in atrophic scarring.
• Bleeding risk (capillary and deep vessel fragility can result in hematoma formation).
• Autonomic dysfunction (postural dysregulation and vasovagal syncope are commonly encountered; when considering device implantation it is critical to exclude these as a cause of syncope).
• Mitral valve prolapse.
• Aortic root dilatation.

Where procedural urgency allows, preoperative imaging may include the following:

• Echocardiography to assess ventricular function, aortic root, and valvular function.
• Cardiac MRI, which may be helpful in ablative procedures to establish the substrate involved, especially if this is uncertain (for example, in undifferentiated cardiomyopathy), as well as providing further assessment of valvular function and aortic size.
• Cardiac CT, which may quantify the extent of coronary disease and aortic root size; CT may also be useful to assess other blood vessel anatomy, especially if a transarterial approach is intended.

With the above information available, an MDT discussion is strongly advocated where clinical urgency allows. If possible, it is ideal if an expert in connective tissue disorders can be involved in the decision-making process.

The interventional approach itself warrants forethought and should be discussed by MDT. As in our case, avoidance of transarterial access is advisable where this is feasible. The risks and benefits of various forms of intraoperative imaging should be weighed up. Where possible, equipment designed to minimize the risk of vascular perforation should be considered.

Finally, further discussion must take place with the patient. A full discussion of the pathology, intended treatment, MDT opinion, and risks and benefits should take place. The patient should be well informed about the increased risks of vascular damage and adverse outcomes, including death. Ultimately, as many EDS subtypes are unknown, the clinician and patient must accept that there is a degree of “unknown” higher risk with invasive procedures.

**Figure 3:**
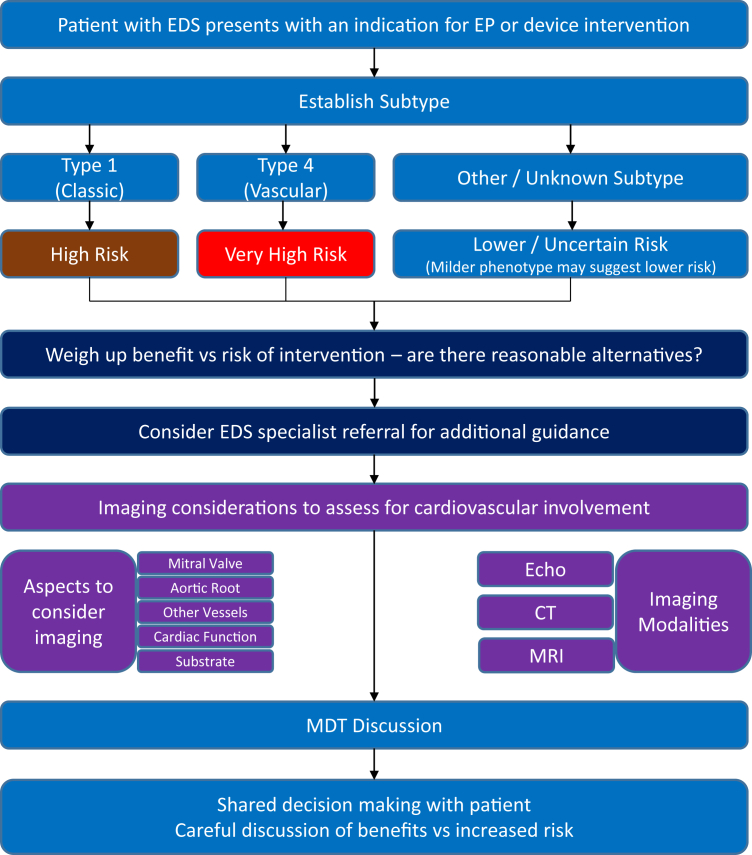
Stepwise approach to interventional procedures in patients with Ehlers-Danlos syndrome (EDS). CT = computed tomography; EP = electrophysiology; MDT = multidisciplinary team; MRI = magnetic resonance imaging.

This approach is summarized in Figure 3.

## Conclusion

Patients with EDS, particularly type 4 (vascular) EDS, are at increased risk of complications from surgical and endovascular procedures. Such interventions should be avoided if at all possible in vascular EDS patients, but may be successful and uncomplicated in those with milder phenotypes. Procedural planning, MDT discussion, and careful consent are essential in managing this complex patient group.

## Appendix Supplementary data

Supplemental Video The first clip presents a Ripple Map during Ventricular Tachycardia, demonstrating endocardial pre-systolic breakout of activation within the scar underneath the inferior mitral annulus. This may represent the endocardial exit of a mid-myocardial/epicardial circuit. The second clip presents a Ripple Map during sinus rhythm, demonstrating the site of latest activation co-located with the site of VT exit. There is a “patchy” channel of delayed activation seen towards this site, either due to low point density or a part mid-myocardial/epicardial course. The final clip reveals the endocardial ablation map (40W, 30-45 sec) homogenizing this channel, hoping to achieve transmural lesions along its course.
